# Transcription Factor EB Is Selectively Reduced in the Nuclear Fractions of Alzheimer's and Amyotrophic Lateral Sclerosis Brains

**DOI:** 10.1155/2016/4732837

**Published:** 2016-06-28

**Authors:** Hongjie Wang, Ruizhi Wang, Shaohua Xu, Madepalli K. Lakshmana

**Affiliations:** ^1^Section of Neurobiology, Torrey Pines Institute for Molecular Studies, 11350 SW Village Parkway, Port St. Lucie, FL 34987, USA; ^2^Florida Institute of Technology, 150 West University Boulevard, Melbourne, FL 32901, USA

## Abstract

Multiple studies suggest that autophagy is strongly dysregulated in Alzheimer's disease (AD) and amyotrophic lateral sclerosis (ALS), as evidenced by accumulation of numerous autophagosomes, lysosomes with discontinuous membranes, and aggregated proteins in the patients' brains. Transcription factor EB (TFEB) was recently discovered to be a master regulator of lysosome biogenesis and autophagy. To examine whether aberrant autophagy in AD and ALS is due to alterations in TFEB expression, we systematically quantified the levels of TFEB in these brains by immunoblotting. Interestingly, cytoplasmic fractions of AD brains showed increased levels of normalized (to tubulin) TFEB only at Braak stage IV (61%, *p* < 0.01). Most importantly, normalized (to lamin) TFEB levels in the nuclear fractions were consistently reduced starting from Braak stage IV (52%, *p* < 0.01), stage V (67%, *p* < 0.01), and stage VI (85%, *p* < 0.01) when compared to normal control (NC) brains. In the ALS brains also, nuclear TFEB levels were reduced by 62% (*p* < 0.001). These data suggest that nuclear TFEB is selectively lost in ALS as well as AD brains, in which TFEB reduction was Braak-stage-dependent. Taken together, the observed reductions in TFEB protein levels may be responsible for the widely reported autophagy defects in these disorders.

## 1. Introduction

Alzheimer's disease (AD) is an irreversible neurodegenerative disorder that presents with progressive intellectual deterioration involving memory, language, and judgment ultimately leading to total dependence on nursing care. It is now estimated that nearly 35.6 million patients are affected by AD worldwide and that about 4.6 million new cases are added each year causing enormous societal and economic burden [1]. Pathologically, AD is characterized by intracellular tau inclusions resulting from tau mutations, extracellular amyloid plaques made up of amyloid *β* peptide (A*β*) derived from amyloid precursor protein (APP), loss of neurons and synapses, astrogliosis, microglial activation, and inflammation [2, 3]. But, as of today, there is no effective therapy for AD and the available treatments can neither reverse nor slow the disease progression as they are not designed to treat the underlying cause of AD. AD has been suggested to have a strong genetic basis with heritability estimates of up to 80% [4]. However, genetic variants in the four well-established genes, namely, APP, presenilin (PS) 1, PS2, and ApoE, and the newly identified nine genetic risk factors for the late-onset AD (LOAD) all together account for less than half of this heritability [5]. Therefore, additional risk genes that contribute to Alzheimer's pathogenesis need to be identified.

Amyotrophic lateral sclerosis (ALS) is a fatal neurodegenerative disorder characterized by muscle wasting and paralysis due to the degeneration of lower and upper motor neurons and their axonal tracts [6]. ALS is usually fatal within 3 to 5 years following diagnosis [7]. About 90% of ALS cases are sporadic (SALS) and the remaining about 10% are caused by mutations in more than fifteen genes and hence classified as familial (FALS) [8]. In the industrialized world, more than one in 500 people will die of the disease [9] and the societal and economic burden is substantial [10, 11]. Nearly 50 randomized clinical trials (RCTs) based on diverse mechanisms have been completed, but none turned out to be successful for ALS [12]. So far, the only available drug riluzole, approved more than 20 years ago, slows the rate of progression and prolongs survival only by 2-3 months [13, 14]. Therefore, novel mechanisms of disease pathogenesis should be identified also for ALS.

Importantly, age is the single major risk factor for AD, ALS, and several other neurological disorders, suggesting that there is an age-associated dysfunction of specific molecular and cellular pathways. In fact, accumulating lines of evidence suggest that autophagy, the pathway that involves delivery of cytoplasmic cargo including aggregated proteins to the lysosomes, is transcriptionally downregulated during normal aging in the human brain [15–17] and even more so in AD [18–21] and ALS [22–24]. Compounded with this deficiency, these disorders have increased production and aggregation of toxic protein aggregates due to mutations in their respective genes that invariably lead to intracytoplasmic accumulation of protein aggregates. In neurons, the two major proteolytic systems that participate in protein turnover and removal of misfolded proteins are the ubiquitin-proteasome system (UPS) and the autophagy-lysosome pathway (ALP). UPS is the first line of defense for normal unfolded protein turnover of monomers but, with the increase in size of the aggregated proteins, UPS can no longer act on them due to narrow pore size of the proteosomal barrel and under such pathological conditions autophagy becomes a vital and predominant pathway to degrade the accumulated proteins. But age-related disorders and aging itself are genetically associated with lysosomal dysfunction [25]. Accordingly, the persistent presence of aggregates that leads to irreversible neurodegeneration and clinical symptoms in these disorders suggests that autophagy response is either dysfunctional or insufficient [26, 27].

Recently, it was discovered that the transcription factor EB [TFEB], a basic helix-loop-helix transcription factor, is a master regulator of lysosome biogenesis [28] which also coordinates autophagy [29], thereby increasing the activity of lysosomal degradative pathways. Moreover, TFEB-induced transcription can stimulate endocytosis [30] and exocytosis [31], which additionally enhances cellular clearance to maintain neuronal proteostasis. Interestingly, TFEB activation has been shown to reduce the accumulation of the pathogenic protein in a cellular model of Huntington's disease (HD) [28] and a mouse model of Parkinson's disease (PD) [32], which was achieved by gene transfer through viral vectors. This suggests that TFEB-induced lysosome biogenesis can effectively clear protein aggregates in neurons which is expected to prevent, stop, or even reverse proteinopathy-induced neurodegeneration and associated behavioral deficits. Since autophagy is the cell's sole mechanism for the bulk degradation of organelles and long-lived proteins [33–35] and since AD and ALS are deficient in this function, we were interested in examining the levels of TFEB protein in the brains of pathologically confirmed cases of AD and ALS.

Here, we quantified the levels of TFEB protein by immunoblots in AD brains with varying degrees of pathology classified based on Braak staging and compared with those of age-matched normal controls. We found selective loss of nuclear TFEB in AD brains in a Braak-stage-dependent manner. We also confirmed reduced nuclear but not cytosolic expression of TFEB in ALS brains using TFEB-specific antibody. Interestingly, we found strong inverse correlation between the extent of pathology and loss of nuclear TFEB in AD.

## 2. Materials and Methods

### 2.1. Chemicals and Antibodies

Protease inhibitor cocktail (cat. # P8340) and dithiothreitol (cat. # D9779), sodium orthovanadate (cat. # 450243), HEPES (cat. # H3375), sodium chloride (cat. # S9888), and ethylenediaminetetraacetic acid (EDTA) (cat. # E9884) were all purchased from Sigma-Aldrich (St. Louis, MO, USA). Microcystin-LR (cat. # 475815) was purchased from Calbiochem-Millipore (Temecula, CA, USA). Nonidet-P40 substitute (cat. # M158) was obtained from Amresco (Solon, OH, USA). Polyclonal TFEB antibody (cat. # 4240) was purchased from Cell Signaling (Danvers, MA, USA) and monoclonal TFEB antibody, clone S1 (cat. # H00007942-M01), was purchased from Abnova (Walnut, CA, USA). Polyclonal lamin A + C antibody (cat. # A01455) was purchased from GenScript (Piscataway, NJ, USA). Mouse monoclonal antibody against actin (cat. # JLA20) was purchased from Developmental Studies Hybridoma Bank (DSHB), University of Iowa (Iowa City, IA, USA). Secondary antibodies such as peroxidase-conjugated AffiniPure goat anti-mouse (code # 115-035-146) and anti-rabbit (code # 111-035-144) IgGs were purchased from Jackson ImmunoResearch Laboratories (West Grove, PA, USA).

### 2.2. Tissue Extraction and Immunoblotting

The AD and normal control (NC) brain tissues (hippocampus) were obtained from the “Harvard Brain Tissue Resource Center” which is supported in part by PHS grant number R24MH068855. The ALS and NC control brain tissues (motor cortex) were obtained from the NICHD Brain and Tissue Bank for Developmental Disorders at the University of Maryland, Baltimore, MD. The lysates were prepared from the AD and ALS brain tissues and the age-matched NC. To prepare the brain homogenates, the brain tissue was rapidly cut into small pieces on ice, weighed, and immediately taken into 1% NP40 buffer (50 mM Tris-HCl, pH 8.0, 150 mM NaCl, 0.02% sodium azide, 400 nM microcystin-LR, 0.5 mM sodium vanadate, and 1% sodium Nonidet P-40) containing complete protease inhibitor cocktail (Sigma, St. Louis, USA) at five volumes' ratio.

As TFEB is known to be present in both the nucleus and the cytosol, we separated cytosolic and nuclear fractions by following methods exactly as used previously in our laboratory [36]. Briefly, the tissues were pestle homogenized in buffer A (10 mM HEPES, pH 7.9, 10 mM NaCl, 0.1 mM EDTA, and 1 mM dithiothreitol plus protease inhibitor cocktail) and centrifuged for 5 min at 3000 rpm in cold. The supernatants were used as cytoplasmic extracts. For nuclear fractions, the pellet was dissolved in buffer C (20 mM HEPES, pH 7.9, 400 mM NaCl, 1 mM EDTA, and 1 mM dithiothreitol plus protease inhibitor cocktail) and vortexed vigorously for 15 min in the cold. The suspension was incubated for 30 min at 4°C under constant shaking. The samples were spun at 14000 rpm for 10 min at 4° C. The supernatants were diluted with buffer D (20 mM HEPES, pH 7.9, and 1 mM EDTA plus protease inhibitor cocktail) at five final volumes and used as nuclear fractions. Protein concentrations from each sample were measured in duplicate by BCA method (Pierce Biotechnology Inc., Rockford, USA). Equal amounts of proteins were loaded into each well and subjected to SDS-PAGE. The proteins were then transferred onto PVDF membranes, blocked with 5% milk, and incubated overnight with primary antibodies followed by one-hour incubation with HRP-conjugated secondary antibodies. The protein signals were detected using Super Signal West Pico Chemiluminescent substrate (Pierce Biotechnology Inc., USA) and normalized to tubulin levels used as loading control.

### 2.3. Statistical Analysis

Immunoblot signals for TFEB in the AD and ALS brains were quantified using publicly available Java-based ImageJ software. The protein levels in NC and AD brains were analyzed by one-way analysis of variance (ANOVA) followed by Dunnett multiple comparisons post hoc test for comparisons among AD and NC brains. To compare between ALS and NC brains, we used Student's paired *t*-test with two-tail *p* value using InStat 3 software (GraphPad Software, San Diego, CA, USA). The data presented are mean ± SEM. The data were considered significant only if *p* < 0.05; *∗∗* indicates *p* < 0.01 and *∗∗∗* indicates *p* < 0.001.

## 3. Results

### 3.1. Striking Reduction of TFEB Protein Levels in the Nuclear Fractions of AD Brains

Because AD brains show severe dysregulation of autophagy [18–21] and since TFEB is the master regulator of lysosome biogenesis which is responsible for regulating autophagy [28, 29], we wanted to verify whether the protein levels of TFEB are altered in AD brains. Therefore, we processed and quantified TFEB protein levels in AD brains with varying degrees of pathology and compared them with age-matched normal controls. The demographics of NC and AD patients are given in Table 1. The age range of NC subjects was 58–86 years and the postmortem interval (PMI) was between 24.08 and 29.18 h. For AD patients, the age range was 71–97 years and the PMI were 6.33–30.83 h.

**Figure 1:**
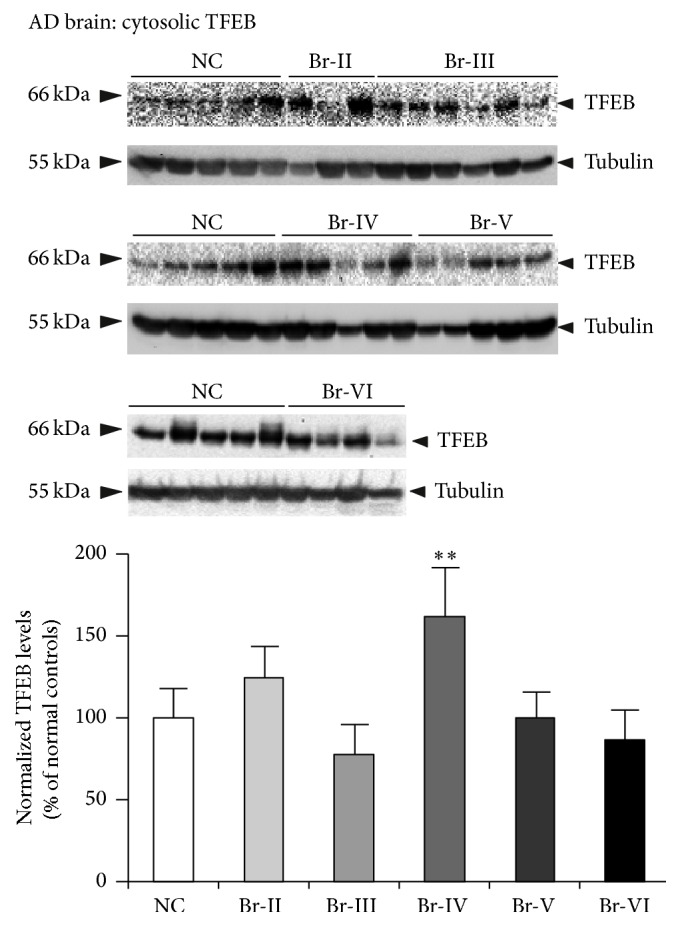
TFEB protein levels are not reduced in the cytosolic fractions of AD brains. Homogenates were prepared from the hippocampus of AD brains classified as Braak stages II to VI or age-matched normal controls (NC), and cytosolic fractions were separated and subjected to SDS-PAGE. Cytoplasmic fractions showed significantly increased normalized (to tubulin) levels of TFEB protein only at Braak stage IV (61%). Tubulin was detected to ensure equal loading of samples and as a marker for cytosolic fractions. ^*∗∗*^ *p* < 0.01 by ANOVA followed by Dunnett multiple comparisons test. Data are mean ± SEM, and “*n*” are indicated on the figure.

**Figure 2:**
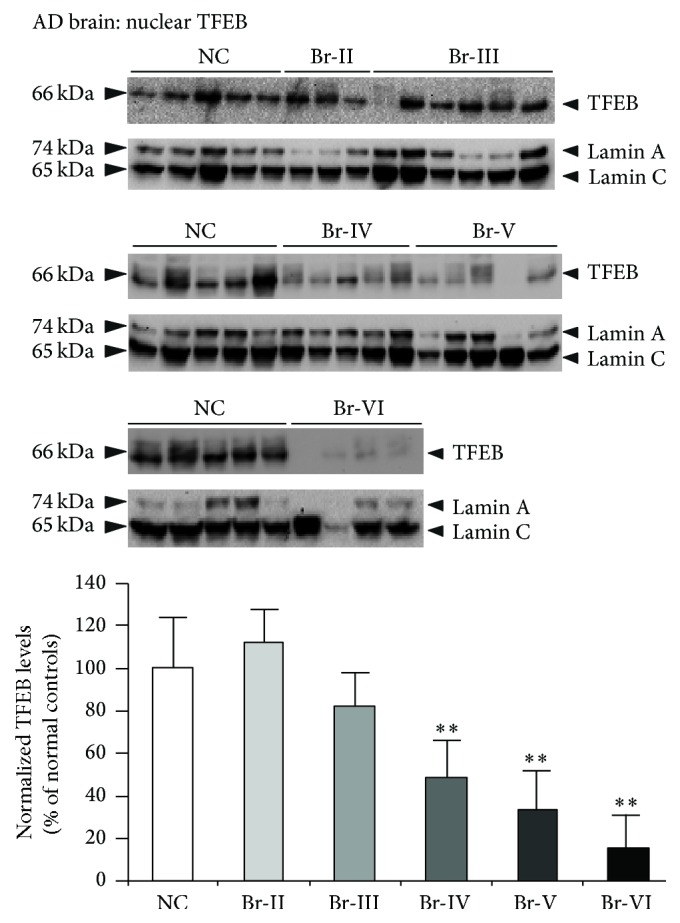
Braak-stage-dependent loss of nuclear expression of the transcription factor TFEB in Alzheimer's brains. Homogenates were prepared from the hippocampus of AD brains classified as Braak stages II to VI or age-matched normal controls (NC) and nuclear fractions were separated and subjected to SDS-PAGE. Nuclear fractions showed robustly decreased normalized (to lamin) TFEB levels starting from Braak stage IV (52%), stage V (67%), and stage VI (85%). Nuclear lamin was detected as a marker of nuclear fractions. ^*∗∗*^ *p* < 0.01 by ANOVA followed by Dunnett multiple comparisons test. Data are mean ± SEM, and “*n*” are indicated on the figure.

Although a majority of endogenous TFEB is found in the cytoplasm under basal conditions, lower levels of TFEB can also be found in the nucleus and a fraction on the lysosomes as well [37]. However, under conditions of stress such as starvation, majority of cytoplasmic TFEB translocate to the nucleus and regulate transcription [37], resulting in biogenesis of new lysosomes [28, 29]. Therefore, we quantified TFEB protein levels in the nuclear and cytosolic fractions of AD brains and compared them with those of NC brains. Positive detection of tubulin in the cytosolic fractions (Figure 1) and lamin A and lamin C in the nuclear fractions (Figure 2) ensured noncontamination of the cytosolic and nuclear preparations. In the cytosolic fractions, normalized TFEB protein levels (to tubulin) were significantly increased by 61% (*p* < 0.01) at Braak stage IV AD brains compared to NC (Figure 1). Though Braak stage II also showed increased trend, it was not significantly different from NC brains (Figure 1). On the contrary, nuclear levels of normalized (to lamin) TFEB protein were consistently reduced starting from Braak stage IV (Figure 2). The reduction was 52% (*p* < 0.01) at Braak stage IV, 67% (*p* < 0.01) at stage V, and 85% (*p* < 0.01) at stage VI (Figure 2). Thus, nuclear TFEB is almost completely lost at Braak stage VI. This also suggests that expression levels of nuclear TFEB are inversely proportional to the extent of tau pathology in AD brains, since Braak staging is based on the extent of tau pathology [38]. Also, alterations in TFEB levels were independent of postmortem interval, sex, or age. Since TFEB protein levels were normalized to that of lamin or tubulin levels, neuronal loss occurring in AD was controlled. Therefore, it is unlikely that reduction in TFEB protein levels in AD brains is due to neuronal cell loss.

### 3.2. TFEB Protein Levels Are Significantly Reduced in the Nuclear but Not Cytosolic Fractions of ALS Brains

**Figure 3:**
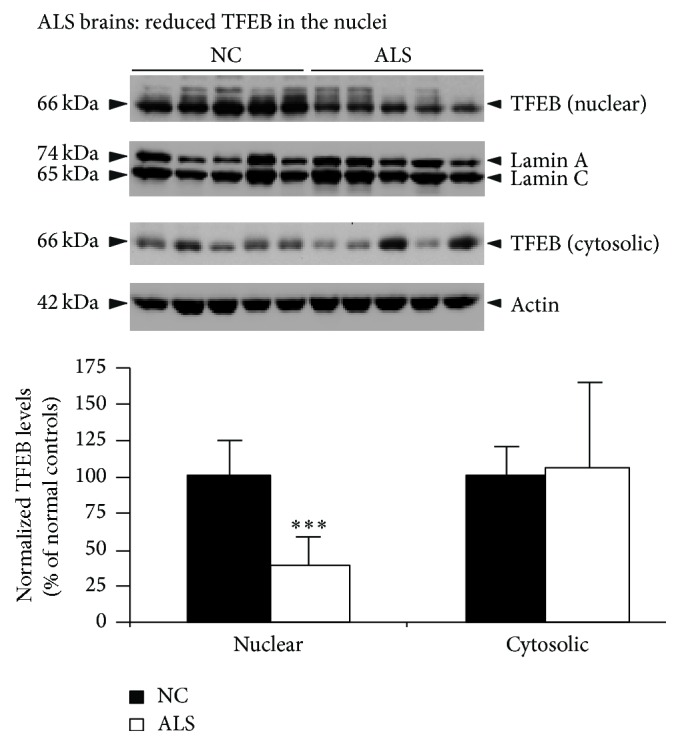
Reduced expression of TFEB protein in ALS nuclear fractions. Lysates of patients brains diagnosed with ALS and NC were prepared, and nuclear and cytosolic fractions were separated as in Figures 1 and 2. Actin and lamin were detected as loading controls and used for normalization of TFEB protein levels. Quantitation by ImageJ revealed significantly reduced (62%) normalized (to lamin) levels of TFEB protein in the nuclear fractions, but no change in the cytosolic fractions. The data are mean ± SEM. ^*∗∗∗*^ *p* < 0.001 by Student's paired *t*-test, *n* = 5 per group.

Since autophagy is also dysregulated in ALS brains [22–24], we obtained ALS and NC brains from NICHD. The demographic details of NC and ALS patients are given in Table 2. The age range of NC subjects was 59–76 years and the PMI was between 3 and 21 h. For ALS patients, the age range was 59–87 years and the PMI were 6–22 h. Both males and females were included in the NC and ALS groups. The motor cortices were subjected to homogenization and separated into nuclear and cytoplasmic fractions as described above for AD brains. Immunoblot detection and quantitation of TFEB protein levels in the cytoplasmic fractions did not reveal any changes in the ALS motor cortex compared to NC. However, nuclear fractions showed a 62% reduction (*p* < 0.001) compared to NC motor cortex (Figure 3). Thus, similar to AD brains, reduction in nuclear TFEB levels in ALS brains suggests possible reduction in TFEB's transcriptional activity.

## 4. Discussion

This study provides the first piece of quantitative data on TFEB protein levels in the AD hippocampus staged and diagnosed based on progression of NFT pathology. The results revealed Braak-stage-dependent alterations in the levels of TFEB protein in the AD brains. This is also the first study to report reduction in TFEB protein levels in the ALS brains. The nuclear fractions of AD revealed striking reductions at later Braak stages, especially showing remarkable reduction at Braak stages V and VI, at which stage there was almost no TFEB protein in the nuclei. On the contrary, TFEB protein levels were increased in the cytosolic fractions at Braak stage IV only, although there was an increased trend at Braak stage II. These results suggest that TFEB protein levels may be selectively lost in the nuclear fractions in a Braak-stage-dependent manner.

Braak staging is based on the extent of spread of intracellular deposits of hyperphosphorylated tau protein in NFT, dystrophic neurites, and neuropil threads [38, 39]. At stages I and II, the NFT pathology is restricted to transentorhinal region of the brain with no evidence of cognitive impairments. It appears that at such prodromal disease states TFEB protein levels are not significantly altered as demonstrated in the present study. With the increase in spread of NFT pathology extending to the hippocampus and other limbic areas of the brain at stage IV, TFEB protein levels start showing significant reductions in the nuclear fractions. Since these Braak stages are also accompanied by mild cognitive impairment (MCI), reduction in TFEB protein levels is correlated with the MCI. The most advanced stage of AD is classified as Braak stages V and VI, by which time the NFT pathology has spread throughout the neocortex and also associated with classical dementia. Reductions in TFEB protein levels in the nuclear fractions were even more robust leading to almost complete absence of TFEB protein at these advanced stages, suggesting that TFEB protein levels are inversely proportional to the extent of NFT pathology and directly correlate with the extent of dementia. Careful analysis also revealed that reduced TFEB in the nuclear fractions is a consequence of more retention of TFEB protein in the soma especially at Braak stage IV. Since no prior study reported TFEB protein levels in AD brains, the present study becomes the first to report and therefore cannot be compared.

In the present study, similar to AD brains, ALS brains also showed marked reductions in nuclear TFEB. TFEB protein levels were also recently shown to be downregulated in both the NSC-34 cell line expressing SOD1^G93A^ mutant protein and spinal motor neurons of transgenic mice expressing SOD1^G93A^ especially at the later stage of the disease [40], suggesting that SOD1^G93A^ mutation affects TFEB expression. Consistent with these findings, TFEB levels were also found to be reduced in both Huntington's disease cell and mouse models [41], implying that reduced expression of TFEB is a key element in the pathogenesis of not only AD and ALS but also other neurodegenerative diseases.

Since TFEB is a transcription factor, it is physiologically relevant only if it is present in the nucleus. Thus, loss of TFEB in the nuclear fractions indicates that TFEB transcription function in AD and ALS brains is compromised. This conclusion is consistent with the overall evidence that autophagy is reduced in AD brains [18–21] as well as ALS as reflected by accumulation of autophagosomes in the spinal cords of both sporadic and familial cases of ALS [22–24], as well as mouse models of ALS [42–45]. Beclin-1, which plays a key role in inducing autophagy by recruiting membrane to the autophagosomes [46, 47], is also reduced in AD brains [48]. Although another protein, LAMP1, a marker of lysosomes, was shown to be increased in AD brains as a whole, interestingly, expression of LAMP1 was inversely correlated with hyperphosphorylated tau deposition in individual neurons with tangles [49]. Moreover, though their numbers are not reduced in AD brains, lysosomes observed in AD brains are aberrant with discontinuous membranes and irregular shape and size. But since TFEB is a master regulator of lysosome biogenesis and autophagy [28–31], loss of TFEB may be primarily responsible for dysregulated autophagy seen in AD and ALS brains.

Reduced TFEB protein may be responsible for accumulation of NFT and amyloid plaques in AD brains and deposition of a variety of mutant protein aggregates in ALS brains. But how TFEB protein levels per se are reduced is unclear at this time. A recent study demonstrated that, in monocytes derived from sporadic AD patients, upregulation of miR128 correlated with impaired degradation of A*β*42 [50]. Interestingly, increased miR128 levels led to reduced TFEB levels in blood cells derived from AD patients [50]. Thus, decreased levels of TFEB protein might be responsible for reduced autophagy in these cells which in turn might be responsible for the reduced degradation of A*β*42. It is also important to note that TFEB expression in the NSC-34 cells increased Beclin-1 and LC3-II protein levels demonstrating that TFEB expression can upregulate autophagy even in a diseased state, which led to increased proliferation and survival [40]. Similarly, more recent studies demonstrated that TFEB overexpression led to marked reduction in the levels of PHF-tau [51] and amyloid plaque burden [52]. Further, in the X-linked SBMA motor neuron disease, caused by block in the autophagosome-lysosome fusion [53], Huntington's disease [54], and Parkinson's disease [55], TFEB could significantly rescue autophagy defects and neurodegeneration. Also, in a recent study, dietary restriction corrected the autophagy defects and partially restored overall neurological symptoms in the dynactin^G59S^ mutant model of ALS, most likely through TFEB activation [56]. Collectively, these observations suggest that reduced TFEB levels and autophagy may be responsible for protein accumulations in these disorders and that by restoring TFEB protein levels it may be possible to reduce protein aggregates and thereby reduce neurodegeneration and restore brain function.

## 5. Conclusion

The current study demonstrated a robust reduction in the expression levels of the transcription factor TFEB in the nuclear but not cytosolic fractions of AD and ALS patients' brains relative to normal controls, which may be responsible for the widely reported defective autophagy markers in these disorders. Thus, by restoring TFEB expression, it may be possible to enhance autophagy in these disorders, thereby rescuing neurodegeneration and its associated behavioral deficits.

## Figures and Tables

**Table 1:** Demographics of normal control subjects and Alzheimer's disease patients obtained from Harvard Brain Tissue Resource Center, used in this study.

Numbers	Diagnosis	Age (y)	Sex	PMI (h)	Tissue
1	Normal control	58	F	26.60	Hippocampus
2	Normal control	77	F	28.00	Hippocampus
3	Normal control	82	M	24.08	Hippocampus
4	Normal control	86	F	29.18	Hippocampus
5	Normal control	82	F	24.42	Hippocampus
6	AD/Braak 2	72	M	6.83	Hippocampus
7	AD/Braak 2	74	M	25.00	Hippocampus
8	AD/Braak 2	81	F	6.33	Hippocampus
9	AD/Braak 3	87	F	22.32	Hippocampus
10	AD/Braak 3	97	F	20.66	Hippocampus
11	AD/Braak 3	77	M	30.83	Hippocampus
12	AD/Braak 3	80	M	23.17	Hippocampus
13	AD/Braak 3	88	F	17.67	Hippocampus
14	AD/Braak 3	89	M	27.17	Hippocampus
15	AD/Braak 4	90	M	24.00	Hippocampus
16	AD/Braak 4	84	F	22.08	Hippocampus
17	AD/Braak 4	83	F	15.63	Hippocampus
18	AD/Braak 4	81	F	22.00	Hippocampus
19	AD/Braak 4	93	F	21.50	Hippocampus
20	AD/Braak 5	83	M	15.90	Hippocampus
21	AD/Braak 5	73	F	29.92	Hippocampus
22	AD/Braak 5	83	M	8.67	Hippocampus
23	AD/Braak 5	80	M	27.50	Hippocampus
24	AD/Braak 5	80	F	9.42	Hippocampus
25	AD/Braak 6	71	M	16.62	Hippocampus
26	AD/Braak 6	84	M	6.66	Hippocampus
27	AD/Braak 6	95	M	15.00	Hippocampus
28	AD/Braak 6	72	M	16.08	Hippocampus

AD: Alzheimer's disease; M: male; F: female; PMI: postmortem interval in hours.

**Table 2:** Demographics of normal control subjects and amyotrophic lateral sclerosis (ALS) patients obtained from NICHD Brain and Tissue Bank for Developmental Disorders, used in this study.

Numbers	Diagnosis	Age (y)	Sex	PMI (h)	Tissue
1	Normal control	61	M	21.00	Motor cortex
2	Normal control	59	M	10.00	Motor cortex
3	Normal control	68	M	19.00	Motor cortex
4	Normal control	72	F	19.00	Motor cortex
5	Normal control	76	F	3.00	Motor cortex
6	ALS	59	M	6.00	Motor cortex
7	ALS	70	M	14.00	Motor cortex
8	ALS	71	F	6.00	Motor cortex
9	ALS	61	F	22.00	Motor cortex
10	ALS	87	F	18.00	Motor cortex

ALS: amyotrophic lateral sclerosis; M: male; F: female; PMI: postmortem interval in hours.
